# New Deferoxamine Glycoconjugates Produced upon Overexpression of Pathway-Specific Regulatory Gene in the Marine Sponge-Derived *Streptomyces albus* PVA94-07

**DOI:** 10.3390/molecules21091131

**Published:** 2016-08-27

**Authors:** Olga N. Sekurova, Ignacio Pérez-Victoria, Jesús Martín, Kristin F. Degnes, Håvard Sletta, Fernando Reyes, Sergey B. Zotchev

**Affiliations:** 1 Department of Pharmacognosy, University of Vienna, 1090 Vienna, Austria; olga.sekurova@univie.ac.at; 2 Fundación MEDINA, Centro de Excelencia en Investigación de Medicamentos Innovadores en Andalucía, 18016 Armilla, Granada, Spain; ignacio.perez-victoria@medinaandalucia.es (I.P.-V.); jesus.martin@medinaandalucia.es (J.M.); fernando.reyes@medinaandalucia.es (F.R.); 3 Department of Biotechnology, SINTEF Materials and Chemistry, N-7465 Trondheim, Norway; Kristin.F.Degnes@sintef.no (K.F.D.); havard.sletta@sintef.no (H.S.)

**Keywords:** *Streptomyces*, secondary metabolite biosynthesis, gene cluster, regulatory gene, deferoxamine, glycoconjugates

## Abstract

Activation of silent biosynthetic gene clusters in *Streptomyces* bacteria via overexpression of cluster-specific regulatory genes is a promising strategy for the discovery of novel bioactive secondary metabolites. This approach was used in an attempt to activate a cryptic gene cluster in a marine sponge-derived *Streptomyces albus* PVA94-07 presumably governing the biosynthesis of peptide-based secondary metabolites. While no new peptide-based metabolites were detected in the recombinant strain, it was shown to produce at least four new analogues of deferoxamine with additional acyl and sugar moieties, for which chemical structures were fully elucidated. Biological activity tests of two of the new deferoxamine analogues revealed weak activity against *Escherichia coli*. The gene knockout experiment in the gene cluster targeted for activation, as well as overexpression of certain genes from this cluster did not have an effect on the production of these compounds by the strain overexpressing the regulator. It seems plausible that the production of such compounds is a response to stress imposed by the production of an as-yet unidentified metabolite specified by the cryptic cluster.

## 1. Introduction

Gram-positive filamentous bacteria belonging to the genus *Streptomyces* are versatile producers of secondary metabolites, many of which have distinct biological activities. Usually, each *Streptomyces* strain produces 1–4 bioactive secondary metabolites in standard laboratory conditions, although it has been shown that varying environmental factors, such as nutrients, temperature, pH, etc., can bring about the production of many more, previously undetected compounds [1]. Recent advances in genome sequencing and analyses helped to explain this phenomenon, as the genome of each *Streptomyces* strain usually harbors 20–50 gene clusters for the biosynthesis of secondary metabolites [2]. In some strains, such gene clusters occupy over 10% of the genome, thus strongly suggesting the importance of corresponding secondary metabolites for the survival and proliferation of *Streptomyces*. Since biosynthesis of secondary metabolites comes at a cost in terms of energy and precursors (drawn from primary metabolites pool), it seems logical that this process is only initiated in response to a particular stimulus. Consequently, it is not surprising that the biosynthesis of secondary metabolites is tightly regulated on several levels, and nearly all gene clusters contain one or more pathway-specific regulators ensuring coordinated expression of biosynthetic genes [3].

The latter feature of secondary metabolite biosynthesis gene clusters has been successfully exploited in order to force the expression of otherwise silent (or cryptic) gene clusters to afford the production of novel compounds [4,5]. Interestingly, it has been shown that some pathway-specific regulators can act pleiotropically on other pathways, suggesting that there exists a cross-talk between them. Huang et al. reported that cluster-specific regulators of *Streptomyces coelicolor* A3(2) can control other secondary metabolite biosynthesis gene clusters and, thus, have pleiotropic actions [6]. Moreover, some pathway-specific regulators can control processes other than the expression of adjacent genes, as demonstrated for the transcriptional activator of the nikkomycin biosynthetic gene cluster of *Streptomyces ansochromogenes*, whose inactivation led, besides abolishing nikkomycin biosynthesis, to reduced sporulation and formation of brown pigment [7].

Recently, we sequenced and analyzed the genomes of two streptomycetes derived from marine sponges collected in the Trondheim fjord (Norway). Interestingly, these isolates were very similar to the terrestrial *Streptomyces albus* J1074, but contained several unique gene clusters for the biosynthesis of secondary metabolites [8]. In this work, a previously uncharacterized putative secondary metabolite biosynthesis gene cluster in marine sponge-derived *Streptomyces albus* PVA94-07 was targeted for activation. Unexpectedly, the recombinant strain, unlike PVA94-07, produced novel deferoxamine glycoconjugates, whose biosynthetic origin could not be traced exactly to the targeted cluster. We describe herein the chemical structure and bioactivity of these compounds and make a proposal on why they are being produced.

## 2. Results and Discussion

### 2.1. Expression of the LysR-Type Cluster-Associated Regulatory Gene in Marine Sponge-Derived S. albus

**Figure 1:**
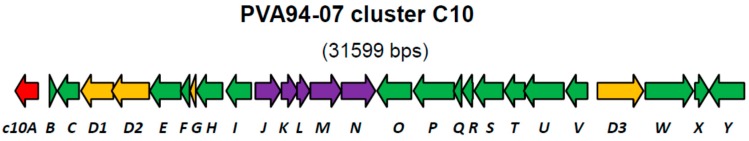
Organization of the C10 putative biosynthetic gene cluster in the genome of *S. albus* PVA94-07. Genes encoding NRPSD-related proteins are in yellow; a LysR-type encoding regulator is in red; and the genes encoding putative modification enzymes are in violet. Genes encoding enzymes with various catalytic activities known to be involved in secondary metabolite biosynthesis are shown in green. A detailed description of the predicted gene function is given in Table 1.

Recently, we reported a draft genome of marine sponge-derived *Streptomyces albus* PVA94-07, which harbors at least 29 gene clusters for the biosynthesis of various secondary metabolites [8]. One of the clusters (C10) encoding discrete adenylation and condensation domains usually associated with non-ribosomal peptide synthetases (NRPS), as well as peptidyl and acyl carrier proteins, appeared to be rare upon database searches and could only be identified in the genome of a marine sediment-derived *Streptomyces* sp. CNQ431. Comparison of the chromosomal fragment encompassing the C10 cluster with a corresponding fragment of *S. albus* J1074 helped to define the borders of this cluster in PVA94-07. Beside the genes encoding NRPSs, which could be involved in the assembly of a peptide scaffold, the cluster encoded several genes encoding putative tailoring enzymes, such as acyltransferases and glycosyltransferases (Figure 1, Table 1).

At the left border of the C10 cluster, a putative regulatory gene *c10A* encoding a LysR-type transcriptional regulator was identified. Regulators of this type contain, besides the DNA binding domain, a ligand-binding site, suggesting that their regulatory function is modulated by a specific chemical signal. LysR-type regulators can be both activators and repressors of transcription [9], and exact roles of these proteins could not be predicted from their amino acid sequences.

Considering the fact that cluster C10 did not contain any other regulatory genes within its presumed borders, it seems plausible that *c10A* is an activator, whose overexpression can lead to the activation of the C10 cluster. With this in mind, the *c10A* gene was PCR-amplified from the genomic DNA of PVA94-07 and cloned under the control of a strong constitutive promoter PermE* in the multi-copy plasmid pUWLoriT [10]. The resulting plasmid was introduced into PVA94-07 via intergeneric conjugation (see Materials and Methods), and the recombinant strain was tested for the production of novel metabolites in comparison with the wild-type strain carrying only pUWLoriT. Shake-flask cultivations were performed in both DNMP, PM4 with soya oil and in NL111 media in order to increase the chance of producing secondary metabolites. Extracts of the cultures were analyzed by HPLC, and the resulting LC-DAD-isoplots were carefully examined to reveal differences in the UV chromatograms of extracts of the control and the recombinant strain overexpressing *c10A*. In extracts of broth cultivated in PM4 supplemented with soya oil, differences between the *c10A* mutant and the control were observed in the UV chromatograms. These differences were not observed in analyses of extracts of the NL111 and DNMP broths. For further investigation of potential new compounds, batch fermentations under controlled conditions were performed in PM4 supplemented with soya oil.

### 2.2. Recombinant PVA94-07 Overexpressing LysR Regulator Produces Novel Derivatives of Deferoxamine

**Figure 2:**
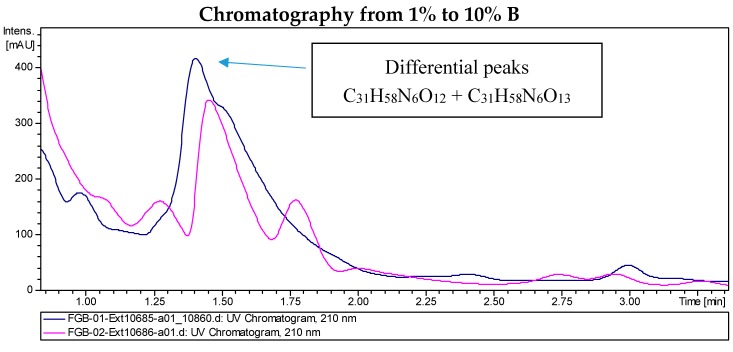

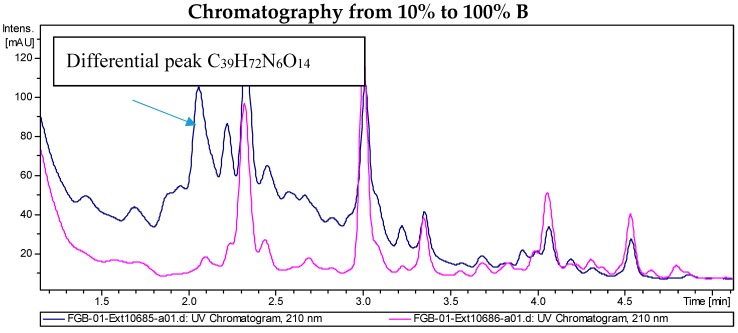
Chromatograms of the extract from *S. albus* PVA94-07 carrying an empty vector (pink) and *S. albus* PVA94-07 overexpressing the *c10A* regulatory gene (blue). Differential peaks and molecular formulae for the corresponding compounds are indicated.

**Figure 3:**
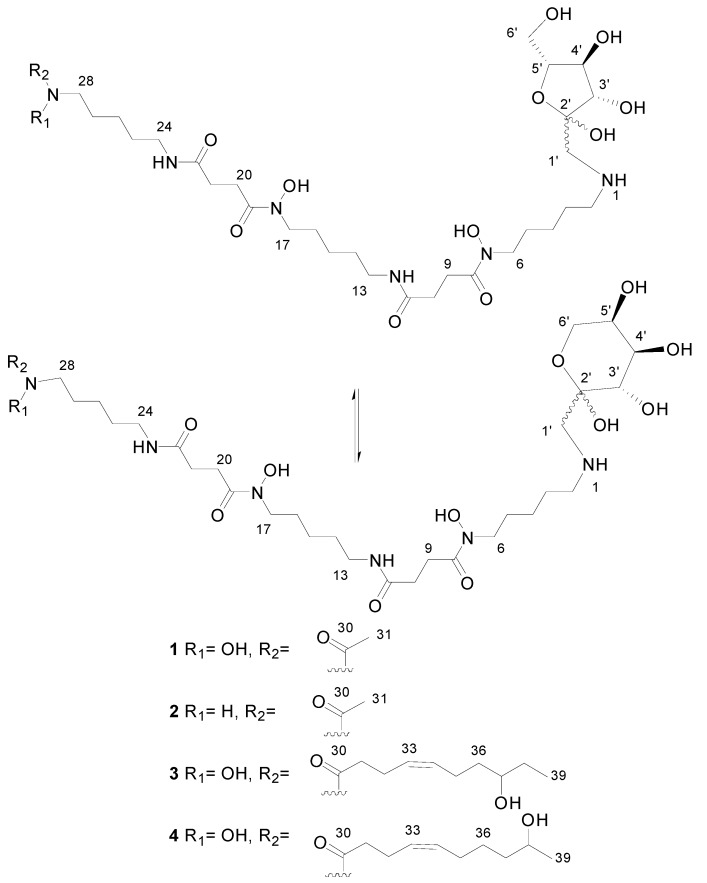
Chemical structures of deferoxamine glycoconjugates produced by genetically-manipulated *S. albus* PVA94-07.

LC/MS analyses of the extracts from PVA94-07 overexpressing *c10A* allowed the identification of accurate masses for four new compounds absent in the control (Figure 2). These included peaks with molecular formulae of C_31_H_58_N_6_O_12_, C_31_H_58_N_6_O_13_, and C_39_H_72_N_6_O_14_ (two different isomers). Subsequently, these compounds were purified using a combination of chromatographic techniques, including low pressure flash chromatography on C18 silica gel and reversed-phase semi-preparative HPLC. Once all of the new compounds were isolated, their structures were determined using HRMS and NMR spectroscopy. Several signals present in the NMR spectra were in agreement with deferoxamine-like derivatives, and in order to facilitate their structural determination, the spectroscopic analysis was started with the compounds having the simpler molecular formulae and ended with the two most complex molecules. LC-HRMS analysis yielded a molecular formula of C_31_H_58_N_6_O_13_ for Compound **1** (Figure 3). Compared with the molecular formula of deferoxamine (C_25_H_48_N_6_O_8_), the difference equals C_6_H_10_O_5_, which matches the composition of a dehydrated hexose, suggesting that the main component could be a glycosylated deferoxamine.

The proton and HSQC-NMR spectra clearly showed the presence of deferoxamine as a structural subunit of the compound. It also showed carbohydrate signals, but their intensity ratio with respect deferoxamine signals was smaller than a 1:1 ratio, suggesting that we were dealing with a mixture of closely-related molecules just differing in the structure of the carbohydrate moiety. The absence of any observable anomeric proton in the spectra suggested the presence of a ketohexose rather than an aldohexose as a subunit of the molecule.

By comparison of the HSQC signals of deferoxamine with those of Compound **1**, it was evident that the proton and carbon chemical shifts at C2 had remarkably changed from 2.93 ppm (^1^H) and 40.3 ppm (^13^C) in deferoxamine to 3.04 ppm (^1^H) and 49.1 ppm (^13^C) in Compound **1**, providing enough evidence that the modification of deferoxamine takes place at position C2, that is the putative glycosylation occurs at the primary amine of deferoxamine. Establishing the nature of the ketohexose was essential at this point, and 2D NMR experiments were analyzed. A broad singlet at 3.2 ppm in the proton spectrum displayed two correlations (of different intensity) in the HSQC spectrum to methylene carbons at 52.4 ppm (weaker) and 53.8 (stronger). Each of these correlations must correspond to a different molecule, and since these carbons do not belong to the deferoxamine moiety, they must be part of the ketohexose unit. Interestingly, the ^1^H and ^13^C chemical shifts of these methylene groups are out of the typical range expected for methylenes in ketohexoses (ca. 3.4–4.0 ppm in ^1^H and ca. 61–65 ppm in ^13^C). Their ^13^C chemical shifts around 53 ppm indicated that these methylenes are not oxygenated, but bonded to nitrogen. Thus, the only possibility was that these methylenes are directly bonded to the amino functionality of deferoxamine. 

The definite evidence for this proposal came from the long-range correlation observed in the HMBC spectrum for these methylene protons (broad singlet at 3.2 ppm) with the carbon at 49.1 ppm (position 2 the in deferoxamine moiety). This observation confirmed that perhaps the ketohexose was not attached to the deferoxamine subunit via the anomeric carbon rendering a glycoside, but via a methylene to render a glycoconjugate where the anomeric carbon remains unblocked to interconvert between the pyranose and furanose forms and also between the two anomeric forms (α and β) for each ring type, providing an explanation for the different intensity ratios observed between the NMR signals of the deferoxamine and the ketohexose moieties. Finally, additional HMBC correlations were observed between the methylene protons (broad singlet at 3.2 ppm) and the anomeric carbons at 96.8 and 103.4 ppm, indicating that the methylene groups directly bonded to the deferoxamine moiety correspond to position 1 in the ketohexose. The carbon at 96.8 ppm was assigned to the anomeric carbon of the ketopyranose form, whereas the carbon at 103.4 ppm corresponded to the anomeric one of the ketofuranose form. Further analysis of the HMBC, HSQC and COSY spectra allowed the identification of the carbons and proton resonance frequencies of the remaining positions of the ketohexose (in each of the pyranose and furanose forms). The values of the ^13^C chemical shifts (Table S2) compared with those reported for free ketohexoses [11] allowed determining the configuration of the monosaccharide as that of fructose. Each ring form of the fructose displayed a preferred anomeric configuration (also determined by ^13^C chemical shifts). The less abundant signals corresponded to the minor anomers. Compound **1** thus corresponds to a novel and striking fructosyl-deferoxamine glycoconjugate. To our knowledge, a similar N-linkage was reported only once before, for the fructose-amino acid conjugate from *Cyperus rotundus* [12].

HRMS analysis established a molecular formula of C_31_H_58_N_6_O_12_ for Compound **2**, differing therefore in the absence of one oxygen atom with respect to Compound **1** and suggesting that both molecules had closely-related fructosyl-deferoxamine glycoconjugate structures. This similarity was evident from the comparison of their ^1^H-NMR spectra. The ketohexose and most of the deferoxamine moiety signals in both compounds were almost identical, and the most remarkable difference was the upfield shift of the methyl signal corresponding to the terminal acetate group, which was moved from 2.10 ppm in Compound **1** to 1.94 ppm in Compound **2**. This difference in chemical shift is in agreement with the replacement of the *N*-hydroxyacetyl group in the structure of **1** by an *N*-acetyl group in **2**.

Compound **3** had a molecular formula of C_39_H_72_N_6_O_14_ according to ESI-TOF HRMS analysis. Such a formula suggests that the deferoxamine and fructose moieties could be present as structural subunits alongside additional features, which would account for the extra number of carbons, hydrogens and oxygens. The ^1^H-NMR spectrum clearly showed the similarity of this compound with **1** and **2**. 

The carbohydrate moiety signals are almost identical in the three molecules. The deferoxamine moiety signals are also present in the spectrum of Compound **3**, but with the remarkable difference of the disappearance of the acetyl methyl singlet. Notably, a number of new signals appears in the spectrum (Table S3), the most significant ones corresponding to two mutually-coupled olefinic protons observed at a δ_H_ above 5.4 ppm, one methine proton at δ_H_ 3.5 ppm (indicating oxygenation at the corresponding carbon), a methyl triplet below 1 ppm and a number of other aliphatic signals partially overlapping with the deferoxamine moiety signals. Comparison of the HSQC spectra of Compounds **1** and **3** confirmed these observations. The absence of the acetyl group and the presence of these other signals indicated that we were dealing with a fructosyl-deferoxamine glycoconjugate structure identical to that of Compound **1** in which the *N*-acetyl group had been substituted by a different acyl chain. 

The problem was thus reduced to the elucidation of the structure of such an acyl moiety. In-depth analysis of all the 2D NMR spectra acquired (COSY, TOCSY, HSQC and HMBC) allowed establishing the structure of the acyl moiety as (*Z*)-8-hydroxydec-4-enyl. The double bound was assigned a Z configuration based on the magnitude of the coupling constant involved between the olefinic protons (ca. 10.5 Hz) and the chemical shifts below 30 ppm of carbons C32 and C35.

Compound **4** was isomeric with **3**, and the same molecular formula was assigned after ESI-TOF analysis. Their NMR spectra clearly showed the expected similarity between both molecules. The most remarkable difference between the ^1^H-NMR spectra (Table S4) was the disappearance of the triplet methyl signal present in the spectrum of **3**, which now appears as a methyl doublet signal at a slightly lower field. The olefinic proton signals appear at an identical chemical shift and with an identical coupling pattern. Thus, the only structural difference is a change in the placement of the secondary hydroxyl group present in the acyl moiety. This hydroxylated methine is now vicinal to the terminal methyl group, explaining its multiplicity as a doublet and also its downfield chemical shift. The comparison of the corresponding HSQC spectra confirmed this conclusion. Compound **4** had therefore a (*Z*)-9-hydroxydec-4-enyl group replacing the acetyl group present in Compound **1**. NMR spectra of Compounds **1**–**4** are given in the Supplemental Materials (Figures S1–S16).

The novel fructosyl-deferoxamine glycoconjugate Compounds **1**–**4** produced by this recombinant *S. albus* PVA94-07 strain resemble the structure of fructose amino acids, also known as Amadori compounds, which are artefact products from the reaction of glucose with amino acids that include a rearrangement step [13]. Such a reaction takes place spontaneously on cooked foods. Since the new fructosyl-deferoxamine glycoconjugates **1**–**4** herein reported are found only in the recombinant strain, they must have a true biosynthetic origin. Their unusual production could involve a unique enzymatic machinery to first conjugate deferoxamine (via its nucleophilic primary amino group) with glucose (catalyzed by a glucosyltransferase using UDP-Glc as the donor) followed by an unprecedented enzyme-catalyzed Amadori-type rearrangement. 

### 2.3. Biological Activity of the New Deferoxamine Glycoconjugates

Two of the new deferoxamine analogues, Compounds **3** and **4** (containing 8 or 9-hydroxydec-4-enoate moieties, respectively), which could be purified in quantities sufficient for bioassays, were tested for anti-microbial activity using the following panel: *Aspergillus fumigatus* (filamentous fungal model), *Candida albicans* (yeast model), *Staphylococcus aureus* MSSA and MRSA (Gram-positive bacteria), *Acinetobacter baumannii*, *Pseudomonas aeruginosa* and *Escherichia coli* (Gram-negative bacterial models). The results obtained showed that none of the two compounds have anti-microbial properties at concentrations ≤16 µg/mL against the microorganisms selected for this panel. However, Compound **4** showed 52%–56% inhibition of *E. coli* at 16 µg/mL, indicating the possibility of improved activity at higher concentrations.

Compounds **3** and **4** were also tested for cytotoxicity using four cell lines (three tumor-derived and one immortalized, non-tumorigenic). None of the compounds exhibited cytotoxic activity on these cell lines in the MTT assays.

### 2.4. Investigations into the Biosynthetic Origin of the New Deferoxamine Glycoconjugates

The deferoxamine biosynthetic gene cluster (C11) in the PVA94-07 genome could be identified ca. 100 kb away from the C10 gene cluster. Considering the results obtained so far, it was tempting to speculate that the new deferoxamine glycoconjugates are produced as a result of an interplay between the enzymes encoded by the C10 and C11 gene clusters. Considering the presence of a sugar on the conjugates and the *c10N* gene encoding a glycosyltransferase, it seemed plausible that the latter enzyme was involved in the biosynthesis of the deferoxamine glycoconjugates. 

To test this hypothesis, the *c10N* gene in PVA94-07 was disrupted, yielding mutant C10ND, which, like the parent strain, did not produce deferoxamine glycoconjugates. This mutant, however, exhibited impaired growth and sporulation, and its construction was repeated, leading to the same result. Since the *c10N* gene is the last one in an apparent five-gene operon (*c10JKLMN*; Figure 1), it is unlikely that its disruption had a polar effect on the downstream gene(s) that is divergently transcribed. This was further confirmed via expression of the *c10N* gene from an autonomously-replicating plasmid in C10ND (see below), which led to the restoration of the wild-type phenotype. The pC10A1 plasmid was then introduced into the C10ND mutant in order to test whether the elimination of the glycosyltransferase C10N would prevent the formation of deferoxamine glycoconjugates in the activated strain. LC-MS analyses of the extracts of recombinant strain C10ND (pC10A1) grown in PM4 and DNMP media identified deferoxamine derivatives **1**, **2** and **3**, suggesting that the *c10N* gene product is not required for the formation of glycoconjugates. Notably, production of Compounds **2** and **3** by the latter recombinant strain was >3-times higher compared to that of PVA94-07 (pC10A).

Still, in view of the phenotype of the *cN10* disruption mutant, it appeared interesting to test the effect of the overexpression of this gene on the metabolite profile of PVA94-07 and C10ND. Therefore, a plasmid carrying the *c10N* gene under the control of the PermE* promoter was constructed and introduced into both PVA94-07 and the C10ND mutant. In addition, the *c10JLKMN* operon was cloned under control of the PermE* promoter and introduced into PVA94-07, yielding strain C10JNex (Table 2). Analysis of the metabolites from the PVA94-07-based recombinant strains detected low-level production of Compound **1**, while the C10ND mutant overexpressing *c10N* was found to produce deferoxamine glycoconjugates **1**, **2** and **3**, same as the C10ND mutant with the overexpressed LysR regulator encoded by the *c10A* gene.

Considering these results and the fact that we could not identify any other gene in the C10 cluster that could encode an enzyme that would conjugate deferoxamine and glucose, it appears possible that the production of deferoxamine glycoconjugates is a response to stress caused by the production of a molecule specified by the C10 gene cluster. Regulatory proteins of LysR family, such as the product of *c10A* gene, usually act as transcriptional activators upon the binding specific ligand and only rarely act as repressors [9]. Upregulation of the *c10JKLMN* operon by C10A may lead to production of a fully-modified molecule that triggers stress response. Considering the predicted products of this operon, such modifications may include acylation and glycosylation. Detrimental effect of the *c10N* inactivation suggests that the non-glycosylated intermediate is toxic to the cell. Considering the effect *c10N* overexpression has on the production of deferoxamine glycoconjugates, the C10N-dependent glycosylation may be also important for the effect the unidentified product of the C10 cluster has on the metabolism of PVA94-07. At the same time, production of Compounds **1**, **2** and **3** by the C10ND mutant with overexpressed *c10A* gene cannot be easily explained. The only hint is the substantially increased amounts of the Compounds **2** and **3** compared to the PVA94-07-based strain, which may suggest that the non-glycosylated molecule triggers a stronger stress response in the form of deferoxamine glycoconjugates biosynthesis. Alternatively, C10A may act as a repressor of at least some of the genes in the C10A cluster. If this were the case, *c10A* overexpression may have shut down the transcription of modification genes, i.e., the *c10JKLMN* operon, leading to the accumulation of unmodified product that caused the formation of deferoxamine glycoconjugates by an unknown mechanism. Such a product, however, would have to be different from that supposedly accumulated by the C10N mutant, since the strain overexpressing the *c10A* gene did not have growth defects. At this point, we can also not rule out the possibility that the *c10A* gene can have a pleiotropic regulatory effect on other biosynthetic gene clusters, thus contributing to the observed phenomenon.

Considering the results obtained so far, the mechanisms behind the attachments of the sugar and the 8/9-hydroxydec-4-enoate moieties in deferoxamine glycoconjugates remain a mystery. Notably, co-cultivation of *Streptomyces coelicolor* with five other actinomycete bacteria led to the production of 12 acyl-deferoxamine analogues with variable acyl chain lengths [16]. The authors speculated that the production of various acyl-deferoxamines expands the range of siderophores with different degrees of solubility, thus modulating the pool of these compounds known as ferrous ion scavengers [17]. The authors also suggest that relaxed specificity of the deferoxamine biosynthesis enzyme, DesC, may be responsible for the incorporation of acyl chains longer than the acetyl or succinyl moieties [18]. It is possible that the same mechanism is functioning during the formation of acylated deferoxamine glycoconjugates in PVA94-07, while the acyl group donors used must be different from those in *S. coelicolor*. Theoretical calculations of logP for Compounds **1** and **3** using ACD/Percepta strongly suggest that **1** can be almost three-times more water-soluble compared to deferoxamine, while the solubility of **3** is rather similar to the latter. Unfortunately, the scarce amount of the compounds prevented experimental testing of this prediction. Interestingly, we have recently observed the formation of deferoxamine glycoconjugates upon an attempt to activate an unrelated gene cluster in another streptomycete (unpublished data). The latter suggests that the formation of these compounds may be a general phenomenon related to stress response. 

## 3. Materials and Methods

### 3.1. DNA Manipulation and Construction of Recombinant Strains

Plasmids and bacterial strains used or constructed during this work are presented in Table 2. 

*Escherichia coli* strains were handled and manipulated as described in Sambrook et al. [19]. *S. albus* PVA94-07 and its derivatives were maintained on SFM medium [17] supplemented with antibiotics, where appropriate. Genomic DNA isolations were done using the Wizard^®^ Genomic DNA Purification Kit (Promega). PCR amplifications were carried out with Q5^®^ High-Fidelity DNA Polymerase (New England Biolabs) using the oligonucleotide primers listed in Table S1 (Supplementary Material), and amplified DNA fragments were purified from agarose gels using QIAquick Gel Extraction Kit (QIAGEN). 

The *c10A* regulatory gene associated with cluster C10 in PVA94-07 was PCR-amplified from the genomic DNA using primers given in Table S1 (Supplementary Materials) and cloned into the replicative vector pUWLoriT under control of the PermE* promoter. The resulting plasmid pC10A1 was introduced into PVA94-07 via conjugation from *E. coli* ET12567 (pUZ8002) as previously described. An internal part of the *c10N* gene from cluster C10 encoding glycosyltransferase was PCR-amplified from the genomic DNA of PVA94-07 using primers given in Table S1 and ligated with the 3.1-kb *Eco*RI-*Hin*dIII fragment of the vector pSOK201 [15]. The resulting construct pC10gtKO was conjugated into PVA94-07 as described above, generating a *c10N*-disrupted mutant C10ND.

The *c10N* gene, as well as the *c10JKLMN* gene operon were amplified from the genomic DNA of PVA94-07 using primers provided in Table S1 (Supplementary Materials) and cloned under control of the PermE* promoter in the multi-copy vector pUWLoriT [10]. The resulting recombinant plasmids, designated pC10Nex and pC10JNex, respectively, were introduced into the PVA94-07 and C10ND mutant via conjugation (see above and Table 2).

### 3.2. Cultivation of *Streptomyces albus* PVA94-07

Pre-cultures of *Streptomyces albus* sp. PVA94-07 clones were cultivated by adding 1 mL glycerol stock to 50 mL 0.5 × TSB medium (Oxoid) supplemented with 20 g/L glucose in 250-mL baffled shake flasks. Each flask was supplemented with 1.5 g of 3-mm glass beads. The cultures were incubated at 30 °C for 16–18 h at 250 rpm (2.5 cm orbital movement). 

The production cultures (shake flasks and fermentors) were inoculated with 3% (*v*/*v*) from TSB-grown pre-cultures. Production in shake flasks was performed in 500-mL baffled shake flasks with 100 mL NL111 medium [20], PM4 medium [21] supplemented with 15 g/L soya oil or DNMP medium (7.5 g/L soytone, 1.6 g/L dry yeast, 21 g/L MOPS, pH adjusted to pH = 6.8) at 30 °C and 200 rpm (2.5 cm orbital movement) for 6 days. Each flask contained 3 g of 3-mm glass beads. Batch fermentations were performed in Applikon 3-litre fermentors with 1.5 L of PM4 medium supplemented with soya oil for 6–7 days. The pH was adjusted to 6.8 prior to inoculation and not controlled during cultivation. Dissolved oxygen, at 20% of saturation, was controlled by automatic adjustment of the stirrer speed. Airflow was regulated manually (0.2–0.4 VVM), dependent on the activity in the fermentor.

### 3.3. Extraction and Qualitative LC-DAD Analysis of Fermentation Broth

Extraction of PVA94-07 cultures was performed as follows: 1 mL freeze-dried culture was extracted with 1 mL of dichloromethane:methanol (2:1) for one hour. Dry matter was removed by centrifugation, the organic solvent in the supernatant was removed by vacuum centrifugation, and the resulting dry matter was re-dissolved in DMSO prior to analysis. Reversed phase liquid chromatography analyses of extracts were performed on an Agilent HPLC system coupled to a diode array detector (Agilent Technologies, Santa Clara, CA, USA). Separation was performed using a Zorbax bonus RP column (2.1 mm × 50 mm, 3.5 µm) at 35 °C and gradient elution with 25 mM formic acid (A) and acetonitrile (B) at a flow rate of 0.3 mL/min. The starting conditions were 5% (B) for 0.5 min, then a linear gradient to 90% (B) during 25 min. 

### 3.4. General Analytical Procedures

Low pressure flash chromatography was carried out using a Teledyne Isco CombiFlash system. Preparative HPLC was carried out in a Gilson GX281 instrument (Gilson Inc., Middleton, WI, USA). NMR spectra were recorded on a Bruker Avance III spectrometer (500 and 125 MHz for ^1^H and ^13^C-NMR, respectively, Bruker, Billerica, MA, USA) equipped with a 1.7-mm TCI MicroCryoProbe™, using the signal of the residual solvent as THE internal reference (^δ^H 3.31 and ^δ^C 49.0 ppm for CD_3_OD). LC-UV-HRMS analysis was performed as previously described [22,23]. Two different gradients were used in these analyses, employing the same solvent system. Solvent A consisted of 10% acetonitrile and 90% water with 1.3 mM trifluoroacetic acid and ammonium formate, and Solvent B was 90% acetonitrile and 10% water with 1.3 mM trifluoroacetic acid and ammonium formate. The first gradient started at 10% B and went to 100% B in 6 minutes, was kept at 100% B for 2 min and returned to 10 % B for 2 min to initialize the system. The second gradient started at 1% B and went to 10% B in 6 min, then to 100% B in 0.1 min, was kept at 100% B for 1.9 min and returned to 1 % B for 2 min to initialize the system. 

### 3.5. Isolation of Deferoxamine Glycoconjugates

A lyophilized culture (38.0 g) of PVA94-07 overexpressing the *c10A* gene was extracted with CH_2_Cl_2_:CH_3_OH 2:1 (4 × 450 mL). The organic phase was evaporated to dryness to afford 13.0 g of dried extract that was subjected to reversed phase low pressure flash chromatography on silica gel (RP18) (70 g C18, 40–60 µm). The column (h = 10 cm, Ø = 3.2 cm) was conditioned with 5% of CH_3_CN in water for 5 min and eluted at 18 mL/min using a linear gradient from 5%–100% of CH3CN in water for 35 min with a final washing step of 20 min at 100% CH_3_CN. A total of 50 fractions of 18 mL were collected. Differential peaks with respect to the PVA94-07 culture previously identified were located in Fractions 11 and 15 of this chromatography by LC/HRMS.

Fraction 11 (18.1 mg) was dissolved in 200 µL of 50% MeOH and further fractionated by reversed phase semi-preparative HPLC (Agilent ZORBAX SB-C8, 9.4 × 250 mm, 5 µm; 3.6 mL/min, UV detection at 210 nm and 280 nm) with a linear gradient of CH3CN in water from 5%–50% over 35 min where fractions containing Compound **1** (1 mg) eluted between 20.5 and 25.5 min and fractions containing Compound **2** (2 mg) eluted at 27 min. Combined dried fractions containing Compound **1** were dissolved in 50 µL of MeOH and further fractionated by reversed phase semi-preparative HPLC (XBridge C18, 10 × 150 mm, 5 µm; 3.6 mL/min, UV detection at 210 nm and 280 nm) with a linear gradient of CH3CN in water from 5%–40% over 35 min where Compound **1** (0.1 mg) eluted between 12 and 15 min. Fractions containing Compound **2** were dissolved in 70 µL of MeOH and further fractionated by reversed phase semi-preparative HPLC (Agilent ZORBAX SB-C8, 9.4 × 250 mm, 5 µm; 3.6 mL/min, UV detection at 210 nm and 280 nm) with a linear gradient of CH3CN in water from 15%–65% over 35 min, where Compound **2** (0.1 mg) eluted at 4 min. Fraction 15 (68.7 mg) was dissolved in 500 µL of MeOH and further fractionated by reversed phase semi-preparative HPLC (XBridge C18, 10 × 150 mm, 5 µm; 3.6 mL/min, UV detection at 210 nm and 280 nm) with a linear gradient of CH3CN in water from 5%–85% over 35 min, where fractions containing Compounds **3** and **4** (4.6 mg) eluted at 13.5 min. Deferoxamine (2.5 mg) eluted at 9.5 minutes from this chromatography. Further purification of fractions containing Compounds **3** and **4** by reversed phase semi-preparative HPLC (XBridge C18, 10 × 150 mm, 5 µm; 3.6 mL/min, UV detection at 210 nm and 280 nm) with a linear gradient of CH_3_CN in water from 10%–28% over 35 min yielded two isomeric components with the molecular formula C_39_H_72_N_6_O_14_ eluting at 27.5 min, Compound **3** (0.3 mg), and 29.0 min, Compound **4** (0.7 mg).

Compound **1**: HRESIMS *m/z* 723.4141 [M + H]^+^ (calcd. for C_31_H_59_N_6_O_13_^+^, 723.4135); for ^1^H- and ^13^C-NMR data, see Table S2.

Compound **2**: HRESIMS *m/z* 707.4177 [M + H]^+^ (calcd. for C_31_H_59_N_6_O_12_^+^, 707.4185);

Compound **3**: HRESIMS *m/z* 849.5195 [M + H]^+^ (calcd. for C_39_H_73_N_6_O_14_^+^, 849.5179); for ^1^H- and ^13^C-NMR data, see Table S3.

Compound **4**: HRESIMS *m/z* 849.5194 [M + H]^+^ (calcd. for C_39_H_73_N_6_O_14_^+^, 849.5179); for partial ^1^H- and ^13^C-NMR data, see Table S4.

### 3.6. Biological Activity Testing

Antimicrobial activities of the deferoxamine glycoconjugates were tested on a panel of microorganisms at Medina Foundation. The following microorganisms were used: *Aspergillus fumigatus* (as the filamentous fungal model), *Candida albicans* (as the yeast model), *Staphylococcus aureus* MSSA and MRSA (as the examples of Gram-positive bacteria), *Acinetobacter baumannii*, *Pseudomonas aeruginosa* and *Escherichia coli* (as the Gram-negative bacterial models), as previously described [24].

Cytotoxic activity was evaluated for Compounds **3** and **4** on the cell lines Hep G2 (hepatocellular carcinoma), MIA PaCa-2 (pancreatic cancer), MCF7 (adenocarcinoma), and THLE-2 (immortalized human liver cells used as an in vitro model for pharmacotoxicological studies) with the MTT colorimetric assay [25].

## Figures and Tables

**Table 1:** Features of the C10 cryptic biosynthetic gene cluster in *S. albus* PVA94-07.

Gene	Protein, aa	Putative Product	Closest Similarity in the Databases
*orf1*	257	Allantoicase	*Streptomyces* sp. MBRL 601, 99%
*c10A*	303	LysR family of transcriptional regulators	*Streptomyces* sp. CNQ431, 99%
*c10B*	97	l-lysine tRNA ligase	*Streptomyces* sp. CNQ431, 98%
*c10C*	281	NADH pyrophosphatase	*Streptomyces griseus* subsp. *griseus* NBRC 13350, 75%
*c10D1*	423	Non-ribosomal peptide synthetase, C-domain only	*Streptomyces* sp. CNQ431, 99%
*c10D2*	491	Non-ribosomal peptide synthetase, A-domain (Phe)	*Streptomyces* sp. CNQ431, 99%
*c10E*	408	Major facilitator superfamily protein	*Streptomyces* sp. CNQ431, 99%
*c10F*	116	Keto-acyl-synthetase 2 motif	*Streptomyces* sp. CNQ431, 99%
*c10G*	76	Peptidyl carrier protein	*Streptomyces* sp. CNQ431, 99%
*c10H*	346	Acetyltransferase (GNAT)	*Streptomyces* sp. CNQ431, 98%
*c10I*	330	Monooxygenase, luciferase-like	*Streptomyces griseus* subsp. *griseus* NBRC 13350, 85%
*c10J*	330	Galactose mutarotase	*Streptomyces* sp. CNQ431, 99%
*c10K*	216	GAT1 peptidase E	*Streptomyces* sp. CNQ431, 99%
*c10L*	168	Acetyltransferase (GNAT) family	*Streptomyces griseus* subsp. *griseus* NBRC 13350, 85%
*c10M*	406	Radical SAM superfamily protein	*Streptomyces* sp. CNQ431, 99%
*c10N*	445	Glycosyltransferase, MGT family	*Streptomyces* sp. CNQ431, 99%
*c10O*	448	Aldehyde dehydrogenase	*Streptomyces* sp. CNQ431, 99%
*c10P*	533	Acyl CoA-ligase	*Streptomyces* sp. CNQ431, 99%
*c10Q*	106	Acyl carrier protein	*Streptomyces griseus* subsp. *griseus* NBRC 13350, 73%
*c10R*	140	Cupin 2 superfamily protein	*Streptomyces griseus* subsp. *griseus* NBRC 13350, 94%
*c10S*	378	Sugar phosphate isomerase	*Streptomyces griseus* subsp. *griseus* NBRC 13350, 83%
*c10T*	271	TIM-barrel enzyme	*Streptomyces* sp. CNQ431, 99%
*c10U*	523	Aspartate aminotransferase	*Streptomyces griseus* subsp. *griseus* NBRC 13350, 88%
*c10V*	282	Clavaminic acid synthetase (CAS)-like	*Streptomyces griseus* subsp. *griseus* NBRC 13350, 82%
*c10D3*	603	Non-ribosomal peptide synthetase, A- and T-domains	*Streptomyces* sp. CNQ431, 99%
*c10W*	627	Hypothetical protein	*Streptomyces* sp. CNQ431, 99%
*c10X*	180	Mrr superfamily DNA binding protein	*Streptomyces* sp. CNQ431, 99%
*c10Y*	460	Glutamyl-tRNA synthetase	*Streptomyces* sp. CNQ431, 99%
*orf2*	76	Hypothetical protein	*Streptomyces albus* J1074, 100%

**Table 2:** Bacterial strains and plasmids used and/or constructed during this study.

**Bacterial Strains**	**Genotype/Phenotype**	**Source/Reference**
*Escherichia coli* DH5α	General cloning host: (*luxS supE44 ΔlacU169, (ϕ80 lacZΔM15) hsdR17*, *recA1*, *endA1*, *gyrA96*, *thi-1*, *relA1)*	BRL
*Escherichia coli* ET125671 (pUZ8002)	Mediates conjugative DNA transfer from RP4 oriT with helper plasmid pUZ8002 (KanR, CmR); methylation deficient (*dam^−^*, *dcm^−^*, *hsdM^−^*)	[14]
*Streptomyces albus* PVA94-07	Wild type, isolated from marine sponge *Phakellia ventilabrum*, Trondheim fjord, Norway	[8]
*S. albus* PVA94-07 C10A1C10A1	Wild type strain harboring C10A1pC10A1 for *c10A* expression	This work
*S. albus* PVA94-07 C10ND	Wild type strain harboring pC10gtKO for *c10N* disruption	This work
*S. albus* PVA94-07 C10Nex	Wild type strain harboring pC10Nex for *c10N* expression	This work
*S. albus* PVA94-07 C10JNex	Wild type strain harboring pC10Jnex for *c10JKLMN* gene operon expression	This work
*S. albus* PVA94-07 (C10ND) pC10Nex	*S. albus* PVA94-07 C10ND strain harboring pC10Nex	This work
*S. albus* PVA94-07 (C10ND) pC10Jnex	*S. albus* PVA94-07 C10ND strain harboring pC10Jnex	This work
**Plasmids**	**Genotype**	**Source/Reference**
pUWLoriT	pIJ101 minimal replicon, Thio^R^, Amp^R^, *RP4 oriT*, *ColEI* replication origin, ermE*p	[10]
pSOK201	pSG5 minimal replicon, Am^R^, *RP4 oriT*, *ColEI* replication origin	[15]
pC10A1	pIJ101 minimal replicon, Thio^R^, Amp^R^, *RP4 oriT*, *ColEI* replication origin, ermE*p, *c10R*	This work
pC10gtKO	pSG5 minimal replicon, Am^R^, *RP4 oriT*, *ColEI* replication origin, an internal part of the *c10N* gene from cluster C10	This work
pC10Nex	pIJ101 minimal replicon, Thio^R^, Amp^R^, *RP4 oriT*, *ColEI* replication origin, ermE*p, *c10N*	This work
pC10JNex	pIJ101 minimal replicon, Thio^R^, Amp^R^, *RP4 oriT*, *ColEI* replication origin, ermE*p, *c10JKLMN* gene operon	This work

## Supplementary Materials

Supplementary materials can be accessed at: http://www.mdpi.com/1420-3049/21/9/1131/s1.
