# Supplementary material for: New Deferoxamine Glycoconjugates Produced upon Overexpression of Pathway-Specific Regulatory Gene in the Marine Sponge-Derived Streptomyces albus PVA94-07
Source: Molecules. 2016 Aug 27;21(9):1131. doi: 10.3390/molecules21091131 (PMC6273380; doi:10.3390/molecules21091131)
Supplement: Supplementary file 1 [file molecules-21-01131-s001.pdf]

Supplementary Materials: New Deferoxamine Glycoconjugates Produced upon Overexpression of Pathway-Specific Regulatory Gene in the Marine Sponge-Derived *Streptomyces albus* PVA94-07

Olga N. Sekurova, Ignacio Pérez-Victoria, Jesús Martín, Kristin F. Degnes, Håvard Sletta, Fernando Reyes and Sergey B. Zotchev

Table S1. Oligonucleotide primers used in this work.

Name	DNA sequence	Target DNA	Construct
C10gt_HX-F	GACGAAGCTTCCGCTGACCGTCGTCGGCTG	gDNA	pC10Nex
C10gt_HX-R	GCAGTCTAGACCAAGTGTGTGACCGTACGTCTGG	gDNA	pC10Nex<br>pC10JNex
C10_SUB_F	GACGAAGCTTGAGCGTTGGGAATCACGCGTGAG	gDNA	pC10JNex
SOK201_F	CGGCACAGGACGAGCGTAATCATGTCATAGCTGTTTCC	pSOK201	pC10gtKO
SOK201_R	GGTAGGCGTCGCTCACAGGTCGACGGATCTTTTCCGCTGC	pSOK201	pC10gtKO
C10GT_F	GATCCGTCGACCTGTAGCGACGCCTACCAGGACG	gDNA	pC10gtKO
C10GT_R	GCTATGACATGATTACGCTCGTCCTGTGCCGCCAGC	gDNA	pC10gtKO
CL10R_F	GACGAAGCTTGCAGTGGTACGTCGCGCTCC	gDNA	pC10R1
CL10R_R	GGACTCTAGAGAACACCGTTCCGCAACCTCTG	gDNA	pC10R1

Table S2. ^{1}H and ^{13}C-NMR data of compound **1** (CD_3OD, 500 MHz).

Position	δ_H (multiplicity, *J* in Hz)	δ_C
2	3.04 (m)	49.1
3	1.73	25.9
4	1.39 (m)	24.1
5	1.68 (m)	26.7
6	3.60 (m)	48.5
8	-	174.2
9	2.76 (t, 6.9)	28.5
10	2.46 (m)	31.1
11	-	174.2
13	3.17	40.0
14	1.53	29.7
15	1.34 (m)	24.6
16	1.64 (m)	26.7
17	3.63 (m)	48.1
19	-	174.2
20	2.76 (t, 6.9)	28.5
21	2.46 (m)	31.1
22	-	174.2
24	3.17	40.0
25	1.53	29.7
26	1.34 (m)	24.6
27	1.64 (m)	26.7
28	3.60 (m)	48.5
30		172.0
31	2.10 (s)	20.0
1′	*β*-pyr 3.23 (br s)	53.8
	α-fur 3.23 (br s)	52.4
2′	*β*-pyr -	96.8
	α-fur -	103.4
3′	*β*-pyr (3.65)	71.4
	α-fur 4.09 (m)	84.4
4′	*β*-pyr 3.79 (m)	71.3
	α-fur 3.97 (m)	77.9
5′	*β*-pyr 3.90 (m)	70.9
	α-fur 4.02 (m)	84.5
6′	*β*-pyr 4.03 (m), 3.71 (m)	65.3
	α-fur 3.78 (m), 3.67 (m)	62.3

^{13}C chemical shifts obtained from the indirect dimension of HSQC and HMBC spectra. ^{1}H chemical shifts in overlapped regions were determined from HSQC cross peaks.

Table S3. ^{1}H and ^{13}C-NMR data of compound **3** (CD_3OD, 500 MHz).

Position	δH (Multiplicity, J in Hz)	δC	Position	δH (Multiplicity, J in Hz)	δC
2	3.04 (m)	49.1	22	-	174.2
3	1.73	25.9	24	3.17	40.0
4	1.39 (m)	24.1	25	1.53	29.7
5	1.68 (m)	26.7	26	1.34 (m)	24.6
6	3.60 (m)	48.5	27	1.64 (m)	26.7
8	-	174.2	28	3.60 (m)	48.5
9	2.76 (t, 6.9)	28.5	30	-	172.0
10	2.46 (m)	31.1	31	2.53 (m)	33.6
11	-	174.2	32	2.37 (m)	23.9
13	3.17	40.0	33	5.41 (m)	129.7
14	1.53	29.7	34	5.43 (m)	131.8
15	1.34 (m)	24.6	35	2.19 (m)	24.6
16	1.64 (m)	26.7	36	1.48 (m)	38.0
17	3.63 (m)	48.1	37	3.47 (m)	73.5
19	-	174.2	38	1.51 (m), 1.44 (m)	31.2
20	2.76 (t, 6.9)	28.5	39	0.96 (t, 7.4)	10.5
21	2.46 (m)	31.1			
1′	β-pyr 3.23 (br s)	53.8			
	α-fur 3.23 (br s)	52.4			
2′	β-pyr -	96.8			
	α-fur -	103.4			
3′	β-pyr (3.65)	71.4			
	α-fur 4.09 (m)	84.4			
4′	β-pyr 3.79 (m)	71.3			
	α-fur 3.97 (m)	77.9			
5′	β-pyr 3.90 (m)	70.9			
	α-fur 4.02 (m)	84.5			
6′	β-pyr 4.03 (m), 3.71 (m)	65.3			
	α-fur 3.78 (m), 3.67 (m)	62.3			

^{13}C chemical shifts obtained from the indirect dimension of HSQC and HMBC spectra. ^{1}H chemical shifts in overlapped regions were determined from HSQC cross peaks.

Table S4. ^{1}H and ^{13}C-NMR chemical shifts of the acyl moiety of compound **4** (CD_3OD, 500 MHz).

Position	δ_H (multiplicity, *J* in Hz)	δ_C
31	2.53 (m)	33.6
32	2.36 (m)	23.9
33	5.42 (m)	129.7
34	5.43 (m)	131.8
35	2.11(m)	28.2
36	1.45 (m)	27.1
37	1.46 (m)	39.8
38	3.74 (m)	68.5
39	1.17 (d, 6.3)	10.5

^{13}C chemical shifts obtained from the indirect dimension of HSQC spectrum. The NMR data for the deferoxamine and fructosyl moieties of compound **4** are identical to those observed for this subunits in compound **3**.

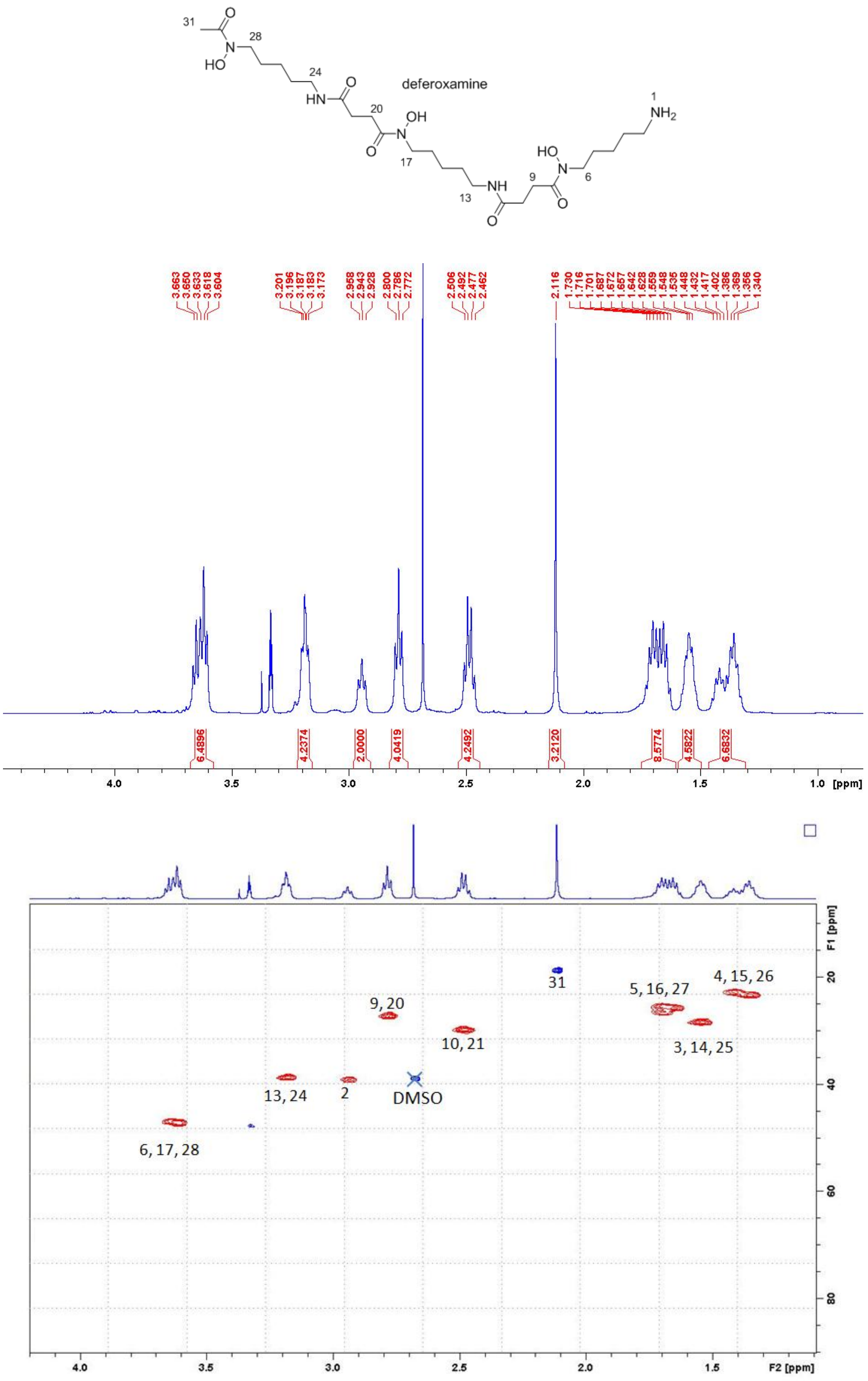

Figure S1. ^{1}H (upper) and HSQC (lower) NMR spectra **4** (CD_3OD, 500 MHz) of deferoxamine isolated alongside compounds **1**–**4**.

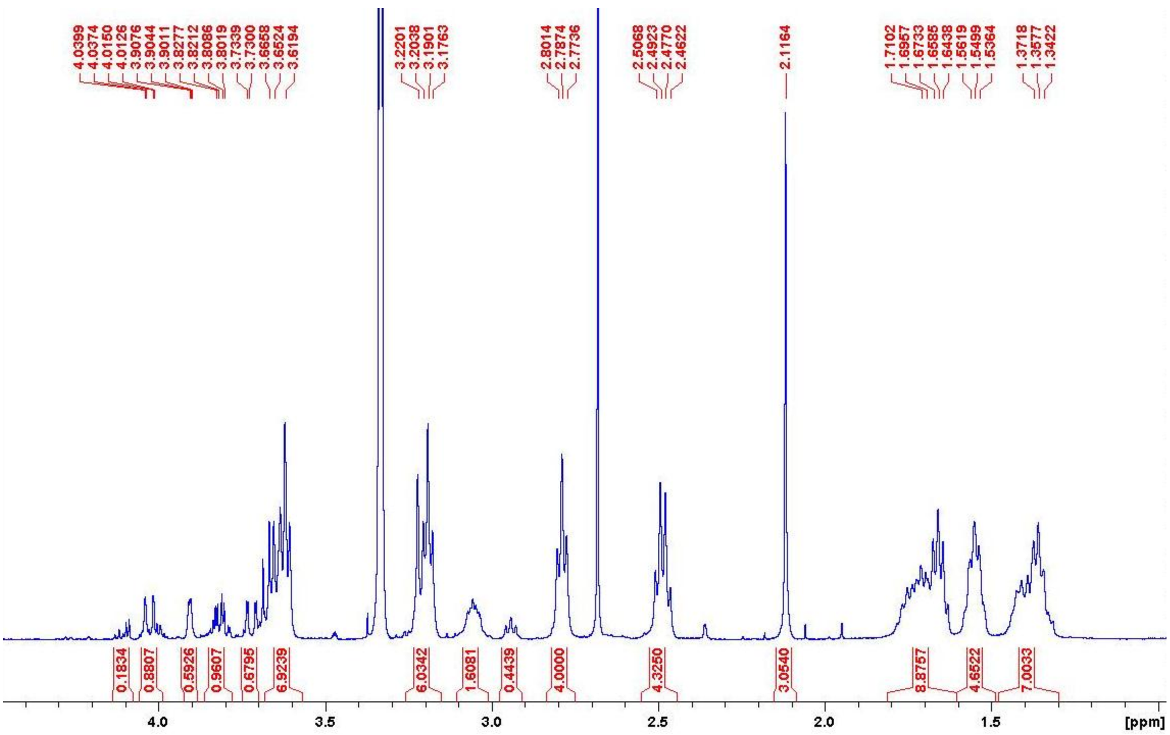

Figure S2. ^{1}H-NMR spectrum of compound **1** (CD_3OD, 500 MHz).

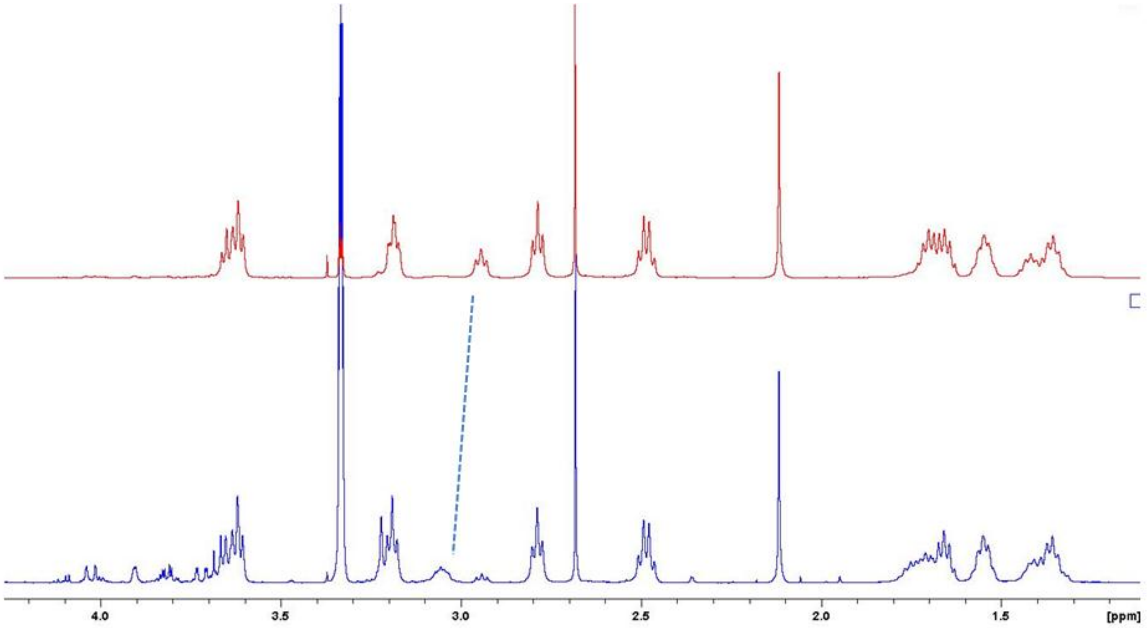

Figure S3. Comparison of the ^{1}H-NMR spectra of compound **1** (blue) and deferoxamine (red).

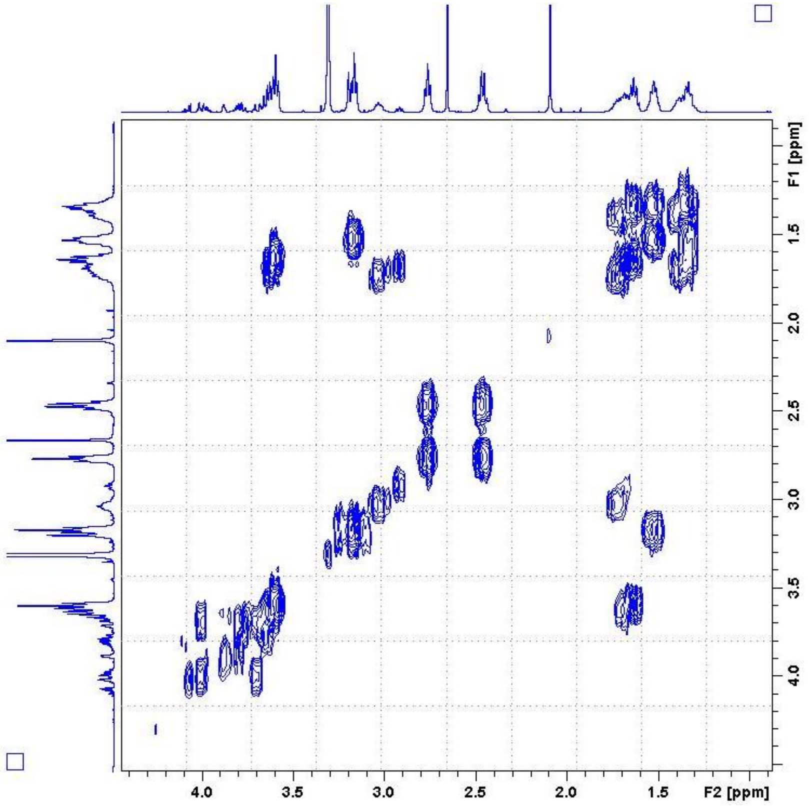

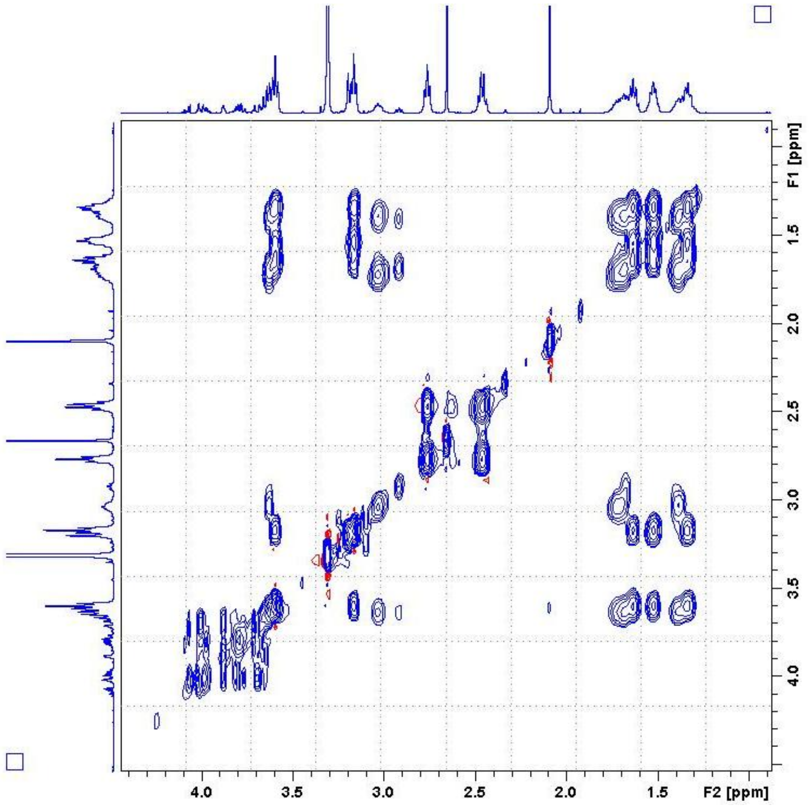

Figure S4. COSY (upper) and TOCSY (lower) spectra of compound **1**.

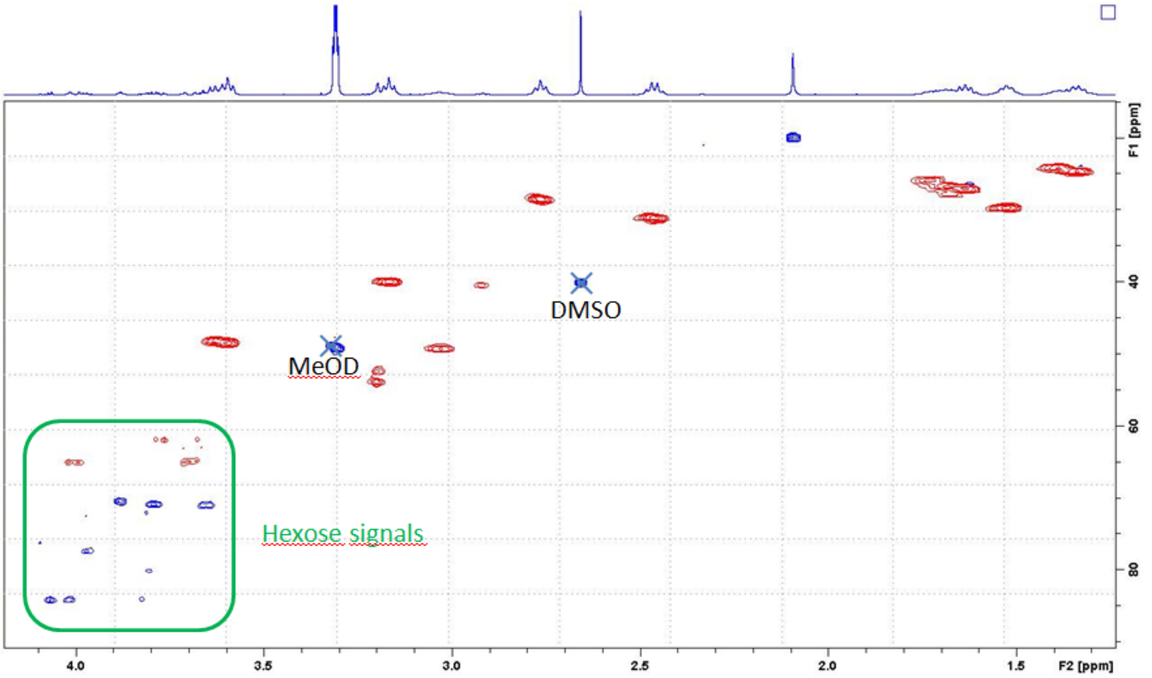

Figure S5. HSQC spectrum of compound **1.** Hexose signals are highlighted.

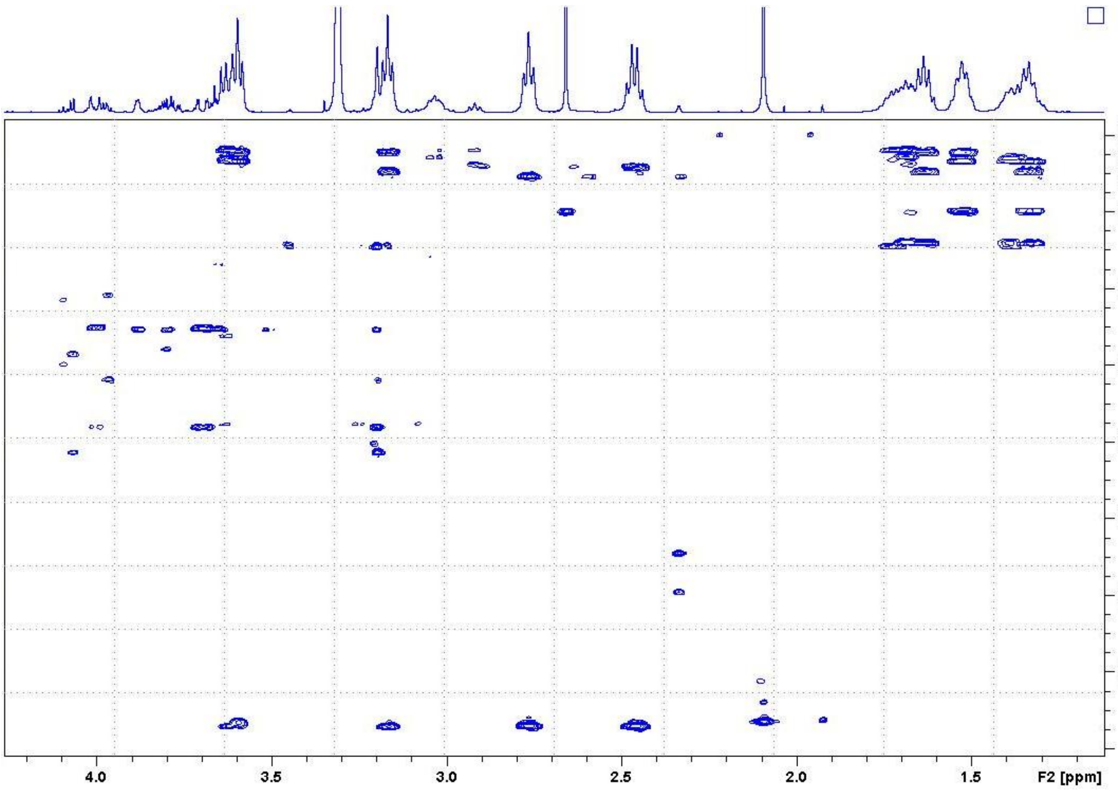

Figure S6. HMBC spectrum of compound **1**.

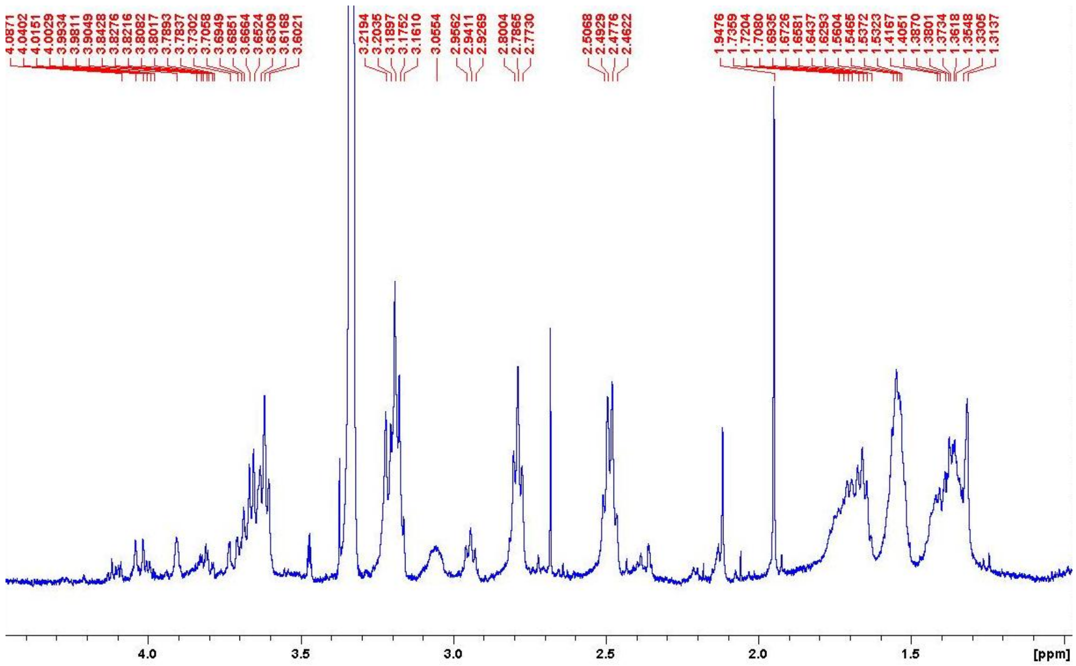

Figure S7. ^{1}H-NMR spectrum of compound **2** (CD_3OD, 500 MHz).

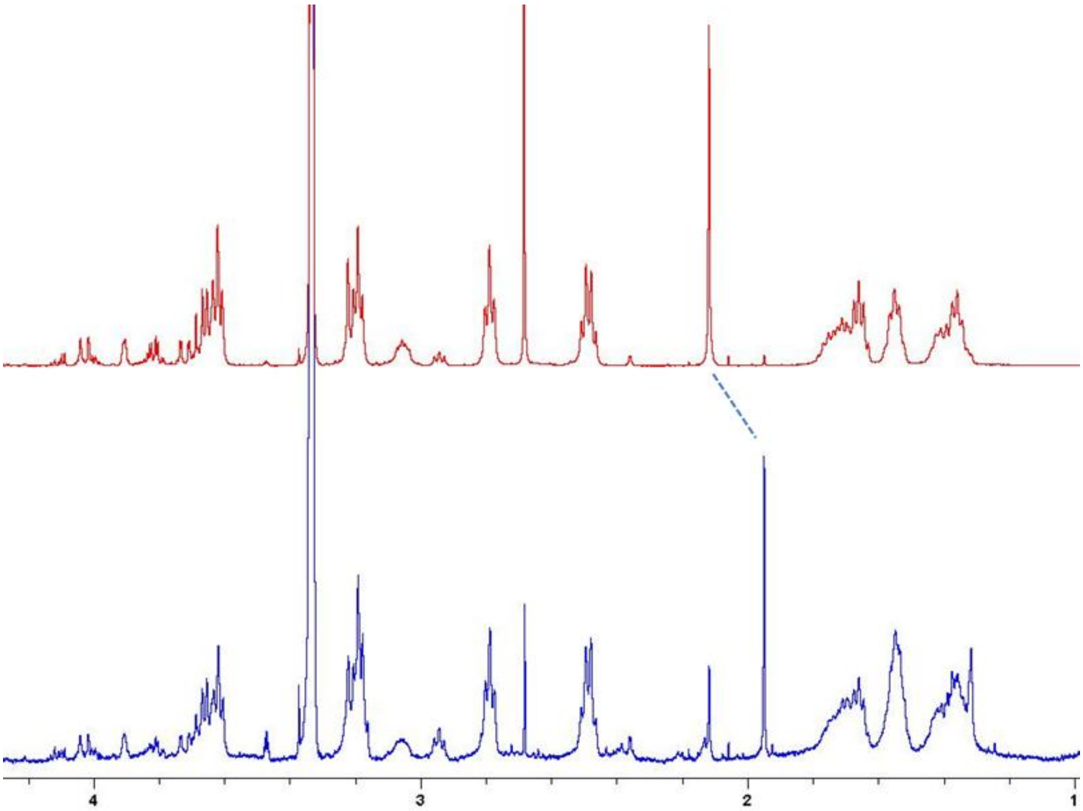

Figure S8. Comparison of the ^{1}H-NMR spectra of compounds **2** (blue) and **1** (red).

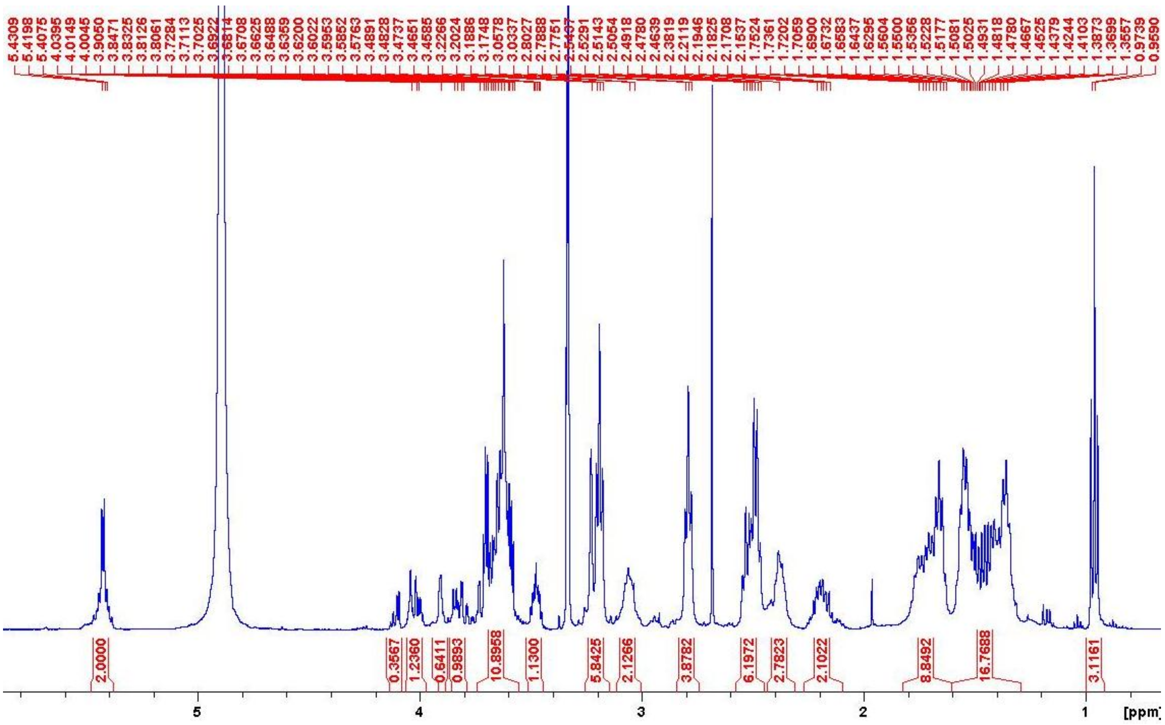

Figure S9. ^{1}H-NMR spectrum of compound **3** (CD_3OD, 500 MHz).

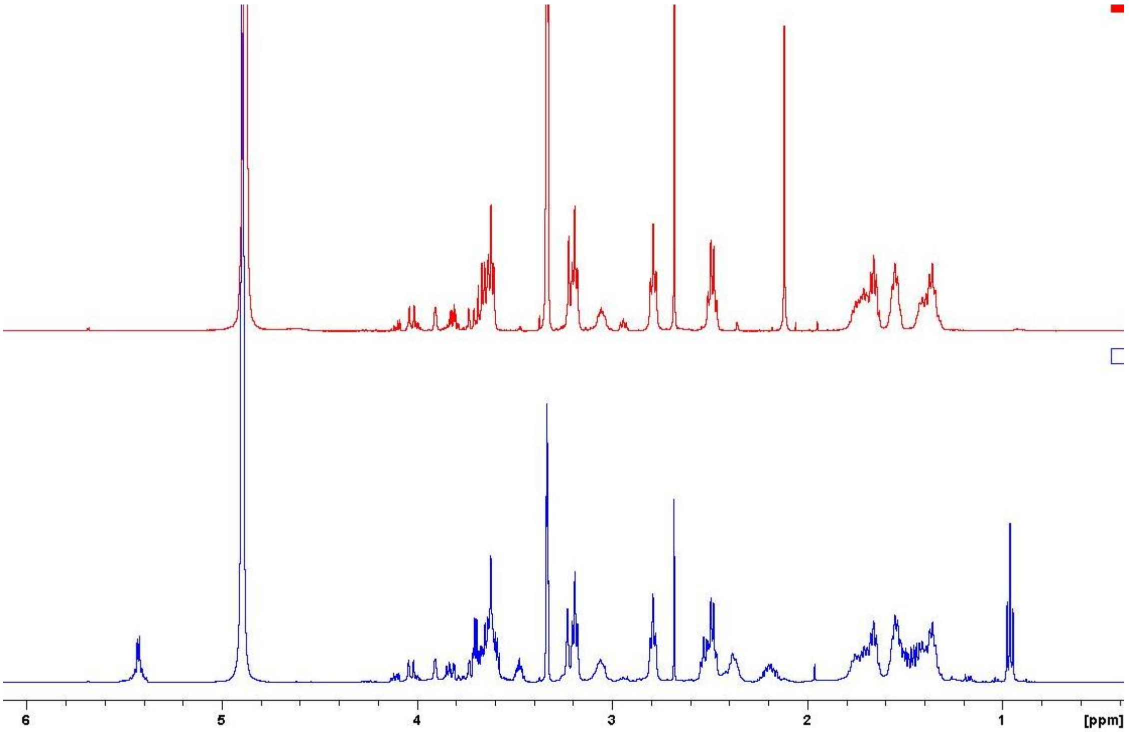

Figure S10. Comparison of the ^{1}H-NMR spectra of compounds **3** (blue) and **1** (red).

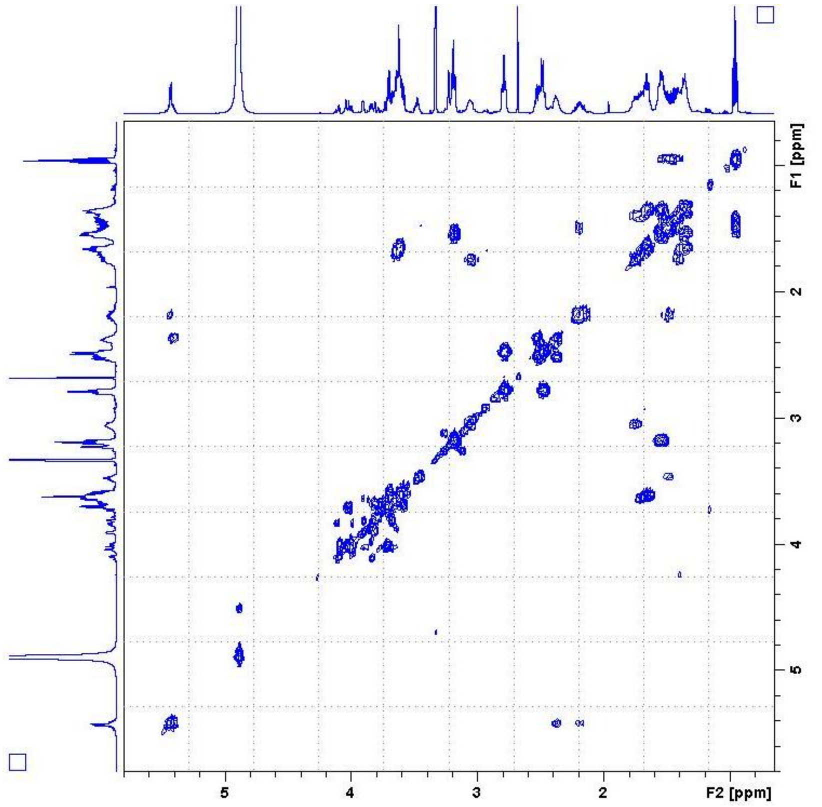

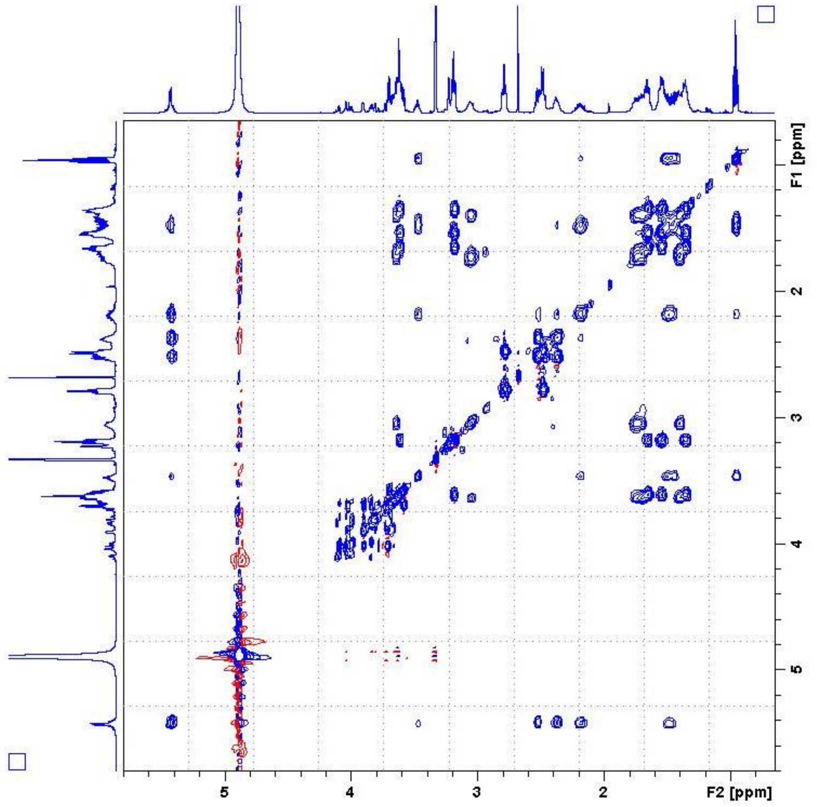

Figure S11. COSY (upper) and TOCSY (lower) spectra of compound **3**.

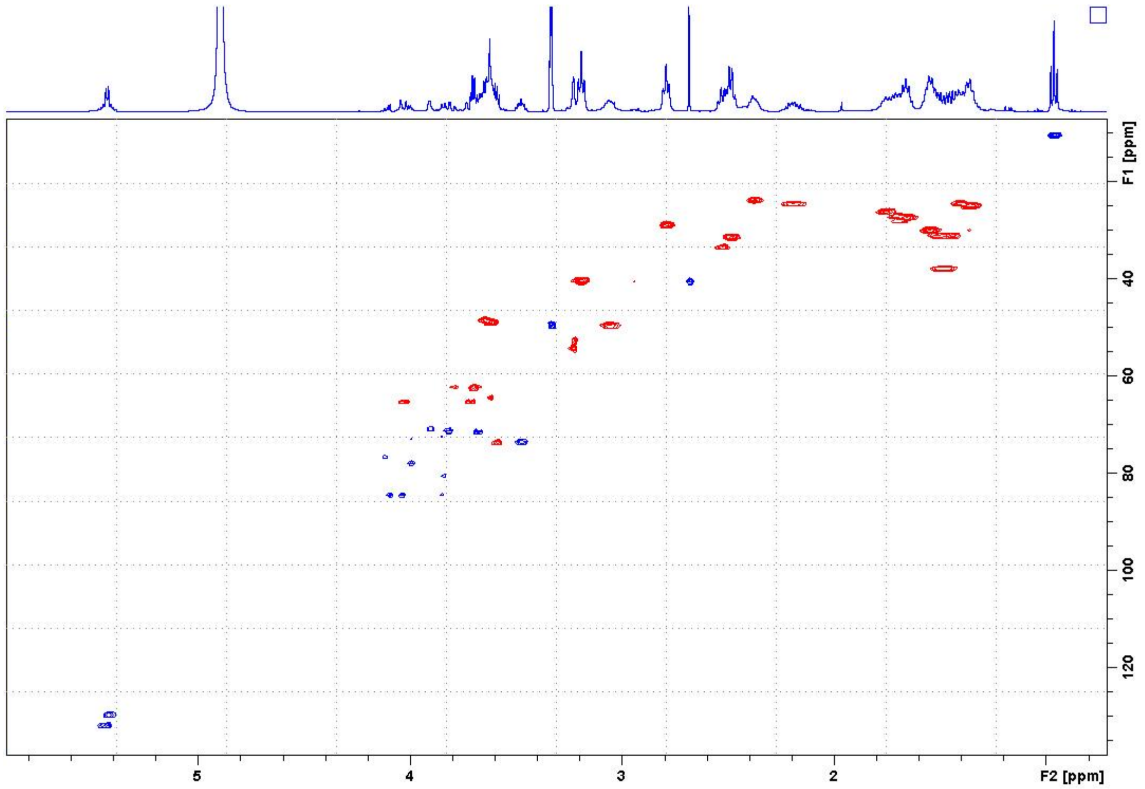

Figure S12. HSQC spectrum of compound **3**.

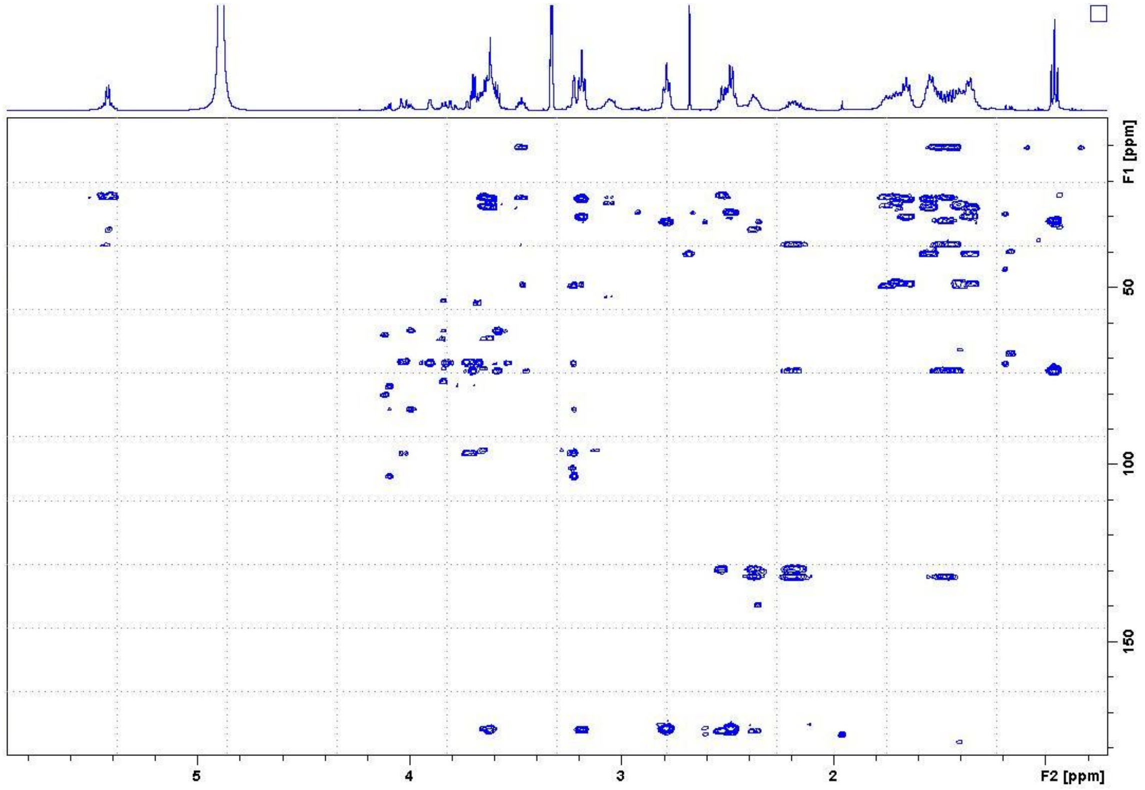

Figure S13. HMBC spectrum of compound **3**.

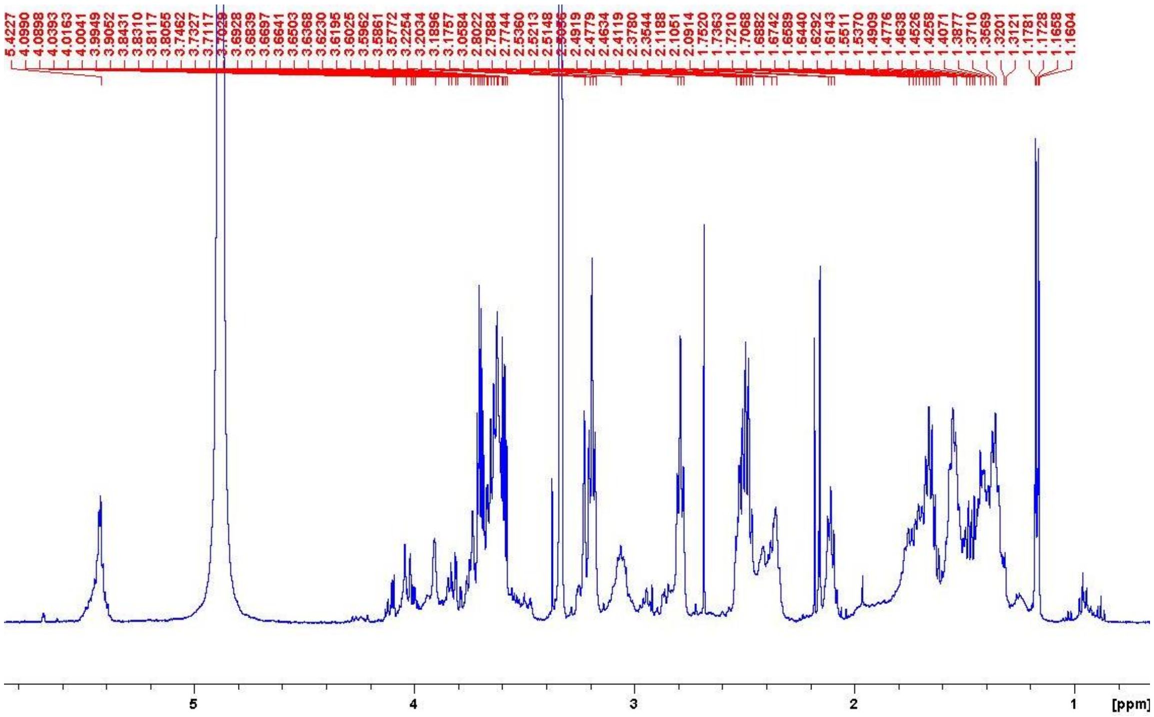

Figure S14. ^{1}H-NMR spectrum of compound **4** (CD_3OD, 500 MHz).

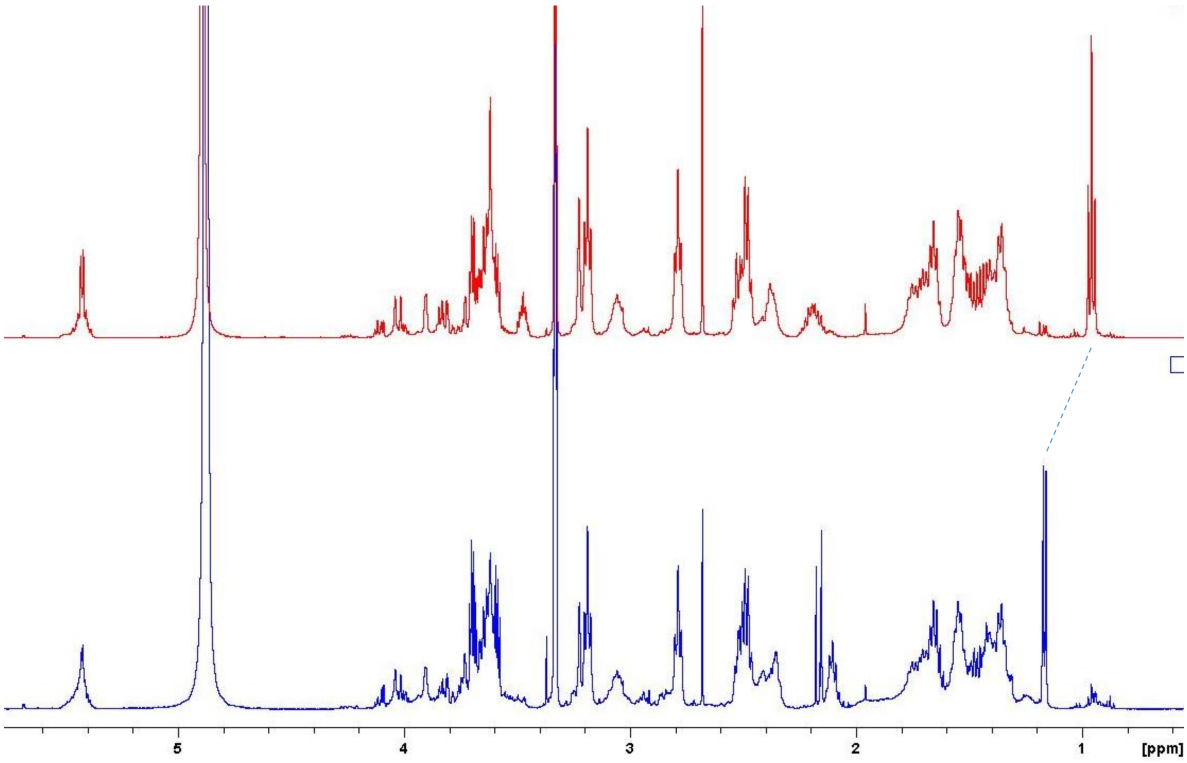

Figure S15. Comparison of the ^{1}H-NMR spectra of compounds **4** (blue) and **3** (red).

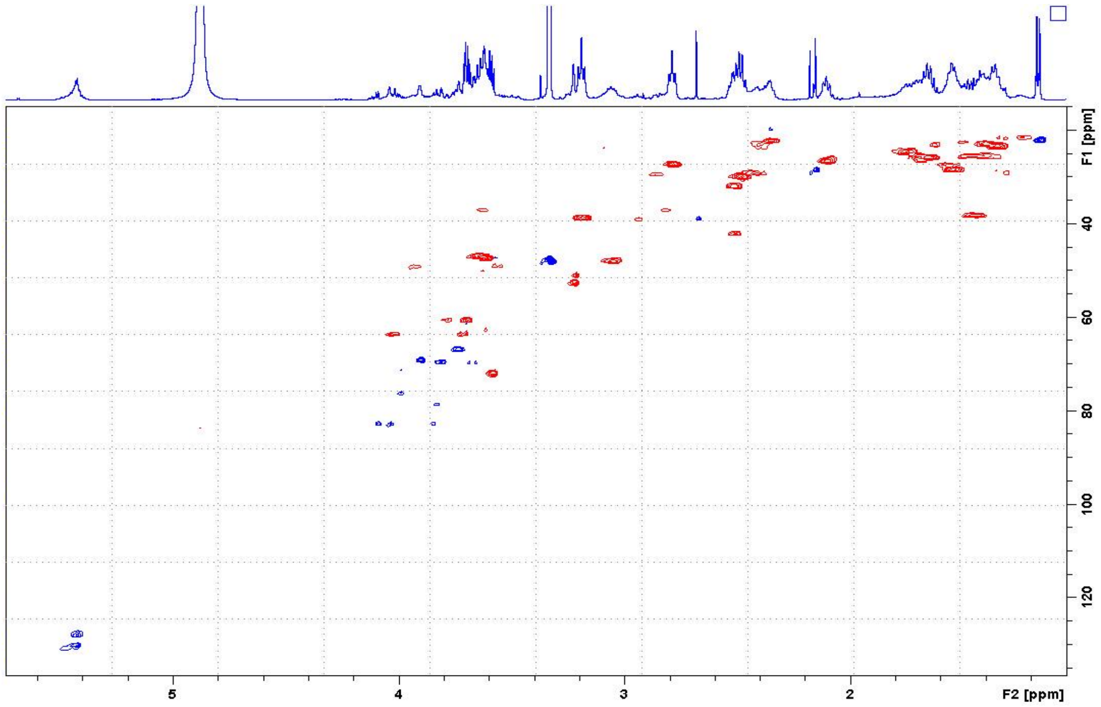

Figure S16. HSQC spectrum of compound **4**.
